# Protocol of the TransformUs Secondary schools program: a type II hybrid implementation-effectiveness trial to increase adolescents’ physical activity and reduce sedentary time in secondary schools

**DOI:** 10.1136/bmjopen-2024-090468

**Published:** 2025-02-10

**Authors:** Ana María Contardo Ayala, Natalie Lander, Emiliano Mazzoli, Anna Timperio, Harriet Koorts, Nicola D Ridgers, Gavin Abbott, David Revalds Lubans, Jo Salmon

**Affiliations:** 1 Institute for Physical Activity and Nutrition (IPAN), School of Exercise and Nutrition Sciences, Faculty of Health, Deakin University, Geelong, Victoria, Australia; 2 School of Health and Social Development, Faculty of Health, Deakin University, Geelong, Victoria, Australia; 3 Alliance for Research in Exercise, Nutrition and Activity, University of South Australia, Adelaide, South Australia, Australia; 4 Centre for Active Living and Learning, College of Human and Social Futures, University of Newcastle, Newcastle, New South Wales, Australia; 5 Hunter Medical Research Institute, New Lambton Heights, New South Wales, Australia; 6 Faculty of Sport and Health Sciences, University of Jyväskylä, Jyväskylä, Finland

**Keywords:** Adolescents, Schools, Exercise

## Abstract

**Introduction:**

Despite the known health and educational benefits of physical activity and the risks of prolonged sedentary behaviour, only one in 10 adolescents globally meet physical activity guidelines, and three-quarters of the school day is spent sitting. *TransformUs*, an effective and cost-effective whole-of-school programme for promoting primary school children’s physical activity and reducing sedentary behaviour, has been adapted for secondary schools (*TransformUs Secondary*). The aim of this paper is to describe the protocol for *TransformUs Secondary* in relation to implementation and scale-up across Australia, and the real-world effectiveness of the intervention on adolescents’ physical activity and sedentary time, as well as sitting time, sleep, well-being and class and school engagement.

**Methods and analysis:**

A type II hybrid implementation-effectiveness trial will be conducted using a mixed-methods design. For the implementation trial, *TransformUs Secondary* will be disseminated via key organisations nationally (eg, state departments of education) and available to all Australian secondary schools (n=1453). Implementation outcomes will be evaluated using the RE-AIM framework (reach, adoption, implementation and maintenance). Data will be collected at the school and teacher levels via the TransformUs website (website analytics), descriptive quantitative surveys, text messages to teachers and qualitative interviews with teachers, students and representatives from key organisations. Descriptive statistics will summarise quantitative data, with regression analyses examining the associations between implementation strategies and outcomes. Implementation levels will be classified as low, moderate or high based on the extent of intervention delivery. Qualitative data will be thematically analysed.

We will assess effectiveness outcomes in 10 Victorian secondary schools using a pragmatic 1:1 waitlist control design. The target sample is 600 Year 7–10 students (12–16 years). Primary outcomes include adolescents’ physical activity and sedentary time (assessed with accelerometry), and secondary outcomes include health (sleep and well-being), class and school engagement (on-task behaviour assessed via classroom observation and school attendance) and sitting time (assessed with posture monitors). Descriptive analyses will summarise students’ demographics, physical activity, sedentary behaviour and engagement, while mixed models will evaluate intervention effects on these outcomes, adjusted for confounders. Additionally, qualitative data will be thematically analysed using deductive and inductive coding in NVivo.

**Ethics and dissemination:**

These trials were approved by the Deakin University Human Research Ethics Committee (2021–269) and by the following education authorities: Australian Capital Territory Education Directorate (RES 2317), New South Wales Department of Education (2022253), Northern Territory Department of Education (20865), Victoria Department of Education (2023_004712), Queensland Department of Education (550/27/2585), South Australian Department of Education (2022–0019), Tasmanian Department of Education (2022–25), Western Australian Department of Education (D23/1152724), and Melbourne Archdiocese Catholic Schools (1232). Results from this study will be disseminated through peer-reviewed journal articles, scientific conferences, summary reports to students and schools and stakeholder meetings.

**Trial registration number:**

Australian Clinical Trials Registration Registry (ACTRN12622000600741).

**STRENGTHS AND LIMITATIONS OF THIS STUDY**

- The implementation trial is being delivered nationally, which is not commonly tested in scale-up studies.
- The 6-month effectiveness trial duration is a limitation as it may take more time for schools to embed the strategies into practice; however, including multiple levels of data collection using several methods (quantitative and qualitative measures) is a strength.
- Device-measured physical activity, sedentary and sitting time, and sleep in the effectiveness trial strengthens confidence in study outcomes.
- The reliance on teacher self-report of programme implementation for both the implementation at scale and effectiveness studies is a limitation; however, engagement with the website resources, professional learning webinars and in-class observations of programme delivery will provide some objective implementation data.
- Strengths include the hybrid implementation-effectiveness trial design that allows for the simultaneous assessment of intervention impact and implementation processes under real-world conditions.

## Introduction

 Physical activity is associated with favourable academic and classroom-related outcomes in adolescents, including improved academic performance,^1^ attention^2^ and executive function.^3^ Conversely, sedentary behaviour has been associated with poorer academic outcomes, such as decreased academic achievement and cognitive functioning.^4 5^ Globally, only 10% of adolescents meet the recommended daily average of 60 min of moderate- to vigorous-intensity physical activity (MVPA) across the week.^6^ While Australia offers some opportunities for sport and physical activity participation,^7^ research consistently shows that physical activity levels among secondary school students remain low, with only 8% of secondary school students meeting physical activity guidelines and more than two-thirds of their waking hours spent in sedentary behaviour.^8^

Schools are an important setting for interventions aimed at increasing physical activity and reducing sedentary time among adolescents. A significant proportion of adolescents’ waking hours are spent in school, and more than three-quarters of this time is spent sitting.^9^ To address these concerns, the WHO released a Schools Physical Activity Toolkit,^10^ which aims to guide school policy-makers and planners in developing and implementing effective policy actions to increase physical activity at schools. Whole-of-school approaches highlighting opportunities to move throughout the school day, including physical education, recess and lunch breaks and during, between and after lessons are recommended.^10^

Despite the importance and need, limited studies have used a whole-of-school approach to promote adolescents’ physical activity. For those few interventions, the outcomes assessed (ie, MVPA) have been variable and largely ineffective in the secondary school context.^11 12^ However, recent evidence suggests that interventions with a strong focus on implementation support (eg, teacher training and intervention resources) have proven effective in increasing MVPA levels among adolescents.^13^ In addition, the interventions that incorporated a range of strategies, such as physical activity sessions, environmental modifications, teacher training, peer support, educational resources and/or active lesson strategies, have shown promise in improving movement behaviours (increased physical activity and reduced sedentary behaviour), cognitive/academic, physical and psychological outcomes in adolescents.^11^

Few effective school-based physical activity programmes have been implemented at scale in secondary schools,^14^ and these initiatives primarily concentrate on enhancing physical education sessions^15^,^17^ and prioritising educational strategies^18^ over whole-of-school interventions that incorporate movement throughout the day. This is despite adolescents spending a substantial portion of their school hours in the classroom,^19^ which remains underused in terms of active learning. There is a need to explore the implementation of whole-of-school interventions that can support physical activity not only during physical education but also within core curriculum areas, such as maths, science and English, and also, there is potential to adapt and test implementation and effectiveness of successfully scaled primary school physical activity programmes in secondary schools.^20^ One whole-of-school physical activity intervention that has proven to be effective and has also been implemented at scale is *TransformUs*; an efficacious primary school programme that integrates movement into core subjects (eg, maths, science and English) as well as environmental changes inside and outside the classroom. *TransformUs* has been shown to reduce and break up sedentary time, increase physical activity and benefit children’s health.^21 22^ It has been offered at scale (via partner organisations) to all primary schools across Victoria, Australia using 14 implementation and scale-up strategies.^23^ Example strategies include the creation of coalitions and networks for programme advocacy and online training to build implementation capacity within schools.^23^ This efficacious whole-of-school primary programme has the potential to be adapted and tested in a secondary school setting.

This paper provides a rationale and description of the *TransformUs Secondary*. The study will include both an implementation trial, aimed at determining the implementation of *TransformUs Secondary* offered at scale to all secondary schools in Australia (Implementation trial) and an effectiveness trial to assess the impact of the intervention on movement behaviours, cognitive/academic outcomes and psychological well-being among adolescents and teachers attending Victorian secondary schools (effectiveness trial).

## Methods and analysis

### Study design

**Figure 1:**
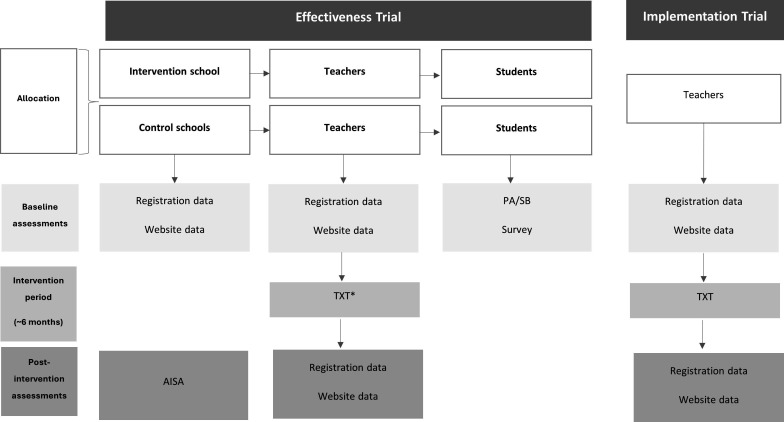
Effectiveness and implementation trial participant and assessment flow diagram. AISA, artificial intelligence-based school audit; PA, physical activity; SB, sedentary behaviour; SOPLAY, System for Observing Play and Leisure Activity in Youth; TXT, text message to teacher’s mobile phone. (*) Intervention teachers only.

A type II hybrid implementation-effectiveness trial^24^ will assess implementation outcomes at the organisational (school and teacher) level and effectiveness outcomes at the individual (student) level, using a mixed methods approach (figure 1). The proposed timeline for the implementation and effectiveness trial can be found in the online supplemental file 1. Online supplemental file 2 contains the Standard Protocol Items: Recommendations for Interventional Trials checklist, relevant to this manuscript. The Implementation research will be conducted and reported in accordance with the requirements of the Standards for Reporting Implementation Studies Statement.^25^

### Program adaptation and piloting

The initial *TransformUs* programme implemented in primary schools has been adapted to the secondary school context through a review of the literature^11^ and a participatory approach. The systematic review identified strategies associated with favourable outcomes for the students (eg, physical activity, sedentary time, academic outcomes and both physical and mental health). These strategies largely aligned with those in *TransformUs* Primary, except for peer support, which was newly developed by researchers and education experts and incorporated into *TransformUs Secondary*. Participatory approach involved interviews with academics (n=8) and key educational organisations (n=4), to determine the barriers and facilitators for the programme and codesign workshops with teachers (n=10), school leader (n=1) and students (n=21). During the workshops, participants offered suggestions to customise *TransformUs Secondary* to fit their secondary school environment addressing the often neglected aspect of the school context in school physical activity intervention research.^26^ The *TransformUs* team further refined these adaptations that were tested in three secondary schools for feasibility in a pilot study. To facilitate scale-up, teacher professional learning sessions, support and all programme resources were adapted to be delivered online. In addition, codesign workshops (n=3) with key education and health organisations across each state and territory of Australia were held so that the programme could be contextualised according to state and territory curricula, priorities and targets. These workshops also provided a platform for sharing knowledge and developing, adapting and refining dissemination and implementation strategies for scaling up *TransformUs Secondary*.

### Intervention

*TransformUs Secondary* includes the delivery of six components: active academic lessons, active breaks,^27^ active homework, health fact sheets, active environments and peer support (see table 1), all underpinned by comprehensive online and onsite teacher professional learning.

**Table 1:** TransformUs Secondary components

Components	Description	Resources
Active academic lessons	Developed by qualified teachers and directly linked to the curriculum, active lessons use incidental and embodied activity within lessons to deliver academic content in an active and engaging way. Incidental activity forms part of the structure of lesson (eg, standing desks, roving stations, collaborating at easels or white boards) and embodied or experiential learning is where the body and/or the movement itself becomes a vehicle for learning.	‘How to’ guides. Active academic lesson plans for English, maths, science, history, geography, psychology, legal studies, civics and citizenship and economics and business.
Active breaks^27^	Short activities that interrupt prolonged periods of sitting in class with short bouts of activity to engage students in learning. The active breaks are used to complement lesson content by using physical and visual reinforcement, introduce, consolidate or summarise lesson content, re-energise and re-engage students in learning, structure the lesson, proactively manage the class and create a positive classroom environment.	‘How to’ guides. Active break examples and plans.
Active homework	Involves integrating movement into homework tasks and is directly linked to the curriculum. Information and home-based activities for parents and students to participate in to engage students in home-based learning and reinforce the importance of adolescents being active.	Active homework examples associated with active academic lessons.
Health fact sheets	These fact sheets to be used by the teachers and students involve physical activity and sedentary behaviour definitions, prevalence and guidelines. The health, behavioural and academic benefits of physical activity. The benefits of physical activity on risk-taking behaviours, mental health and well-being and sleep. The benefits of physical activity for nutrition.	Health fact sheets.
Active environments	Environmental changes such as active indoor/outdoor environment, active equipment, teacher encouragement (champion) and active school policies. Also includes signage, equipment, facilities, resources, policies and teacher support (eg, how to guides) to promote, foster and facilitate physical activity inside the classroom and during recess and lunchtime.	‘How to’ guides. Downloadable templates including champion teacher position description, school policy template, active classroom configuration and active classroom signage.
Peer support	Structured peer mentoring offers a practical, adaptable and customised approach to enhancing physical activity within a secondary school environment. By employing trained high school mentors to facilitate behaviour change in their younger peers, this structured approach incorporates informal support mechanisms. This includes students actively supporting their peers, serving as role models for active behaviours and advocating for or becoming champions of change. Furthermore, the initiative involves dedicated student leaders who actively contribute to decision-making processes related to school-based activities.	Peer support ‘How to’ guides and examples.

### Implementation support strategies

Programme delivery will be supported by teacher professional learning that will be delivered online via webinars or the *TransformUs* website (https://transformus.com.au) for the implementation trial and face-to-face by a research team member (education lead) for the effectiveness trial. Teachers will receive access to the *TransformUs* website resources to aid with programme delivery, including Australian curriculum-aligned lessons, active break resources and active environment guidance; ‘How to’ and ‘Getting started ’guides; frequently asked questions and external support from the research team via email/phone (eg, accessing materials).

The *implementation support strategies* for schools will involve the following: using multiple dissemination and promotion routes/channels; providing an online platform for programme materials and training; enabling implementation flexibility and contextual adaptation (eg, non-prescriptive approach to implementation); enabling both ‘top-down’ and ‘bottom-up’ programme adoption (eg, individual teachers may register and implement the programme or principals/school leaders may register the whole school); using existing school resources, equipment and facilities in the delivery system; alignment with existing state-level initiatives and guidelines; an online implementation support network (eg, via TransformUs webinars); provision of resources to support implementation processes and sustainability (eg, ‘How to’ guides) and monitoring and evaluation to adjust scaling strategy feedback to support schools (eg, follow-up monitoring with partner organisation dissemination activities).

### Patient and public involvement

At what stage in the research process were patients/the public first involved in the research and how?

*TransformUs Secondary* has been developed through a participatory approach involving key education organisations (ie, department of education, teacher professional development organisations, a principals’ association), teachers, school leaders and students (including interviews and workshops) and has been piloted in three secondary schools in Victoria, Australia. The partners were closely engaged in adapting the programme for scale-up (participating in an implementation and dissemination workshop, *TransformUs Secondary* webpage launch and webinars) for the implementation/effectiveness trial.

How were the research question(s) and outcome measures developed and informed by their priorities, experience and preferences?

The development of research questions and outcome measures was informed by the priorities of government education departments in terms of student education outcomes and well-being (including physical activity) and were framed based on the experience of the research team and the preferences of the key organisations that participated in the adaptation phase.

How were patients/the public involved in the design of this study?

Teachers, students, teacher professional development organisations and key education organisations were actively involved in the adaptation of the intervention for the implementation and effectiveness trials.

How were they involved in the recruitment to and conduct of the study?

Education and health partners nationally are playing a crucial role in disseminating the programme, recruiting schools and teachers via their regular channels of communication, and using a toolkit codeveloped by the research team and organisations that participated in the implementation and dissemination workshops. This toolkit consists of a suite of resources to use across each organisation’s communication platforms including social media, newsletter content and flyers. The goal of this toolkit is to help organisations raise awareness of the programme, drive teachers to the website, registration and support for schools and teachers to implement the programme.

Were they asked to assess the burden of the intervention and the time required to participate in the research?

The burden and time required for participation were assessed through the *TransformUs Secondary* pilot study and have also been reported for TransformUs Primary.^28^ This information was critical for designing and adapting the current project.

How were (or will) they be involved in your plans to disseminate the study results to the participants and relevant wider patient communities (eg, by choosing what information/results to share, when and in what format)?

Education partners will continue to play a role in disseminating the study results to participants and relevant communities. They will contribute to sharing information through various platforms such as social media, websites, newsletters, email distribution lists and teacher education professional learning events. Results will also be directly disseminated to all schools and teachers registered in the programme.

### Implementation trial

*TransformUs Secondary* will be freely available to all (n=1453) secondary schools and associated teachers across Australia via the *TransformUs* website during the trial. Schools in the state of Victoria will only be offered the programme once effectiveness trial recruitment (and baseline measures) in Victoria is close to completion. Secondary schools and/or teachers will be able to register with *TransformUs Secondary* online in order to (a) access and use the programme without any research component or (b) participate in the research whereby they complete a baseline survey, then access and use the programme and after 6 months complete a further survey about the implementation of the programme.

### Participants and recruitment

Representatives from the existing *TransformUs* primary school education and health networks in Victoria (eg, government departments of education and health, teacher and principal associations), as well as new partner organisations nationally, will be invited to participate in a series of state-based online partnership meetings with the research team. These meetings will describe the programme rationale and research and request assistance from these organisations with dissemination through advertising, promotion and recruitment across their networks using materials provided by the team (eg, email/social media post). This promotional material will contain a link to the *TransformUs* website registration form, which interested school staff (eg, school principals, school leaders and teachers) will complete. The registration form asks registrants if they would like to be involved in the research component for eligible schools (eg, government and independent schools across Australia, and Melbourne Archdiocese Catholic Schools). If ‘yes’ is selected, registered school staff will be provided with a link to the plain language statement and consent form in the Research Electronic Data Capture (REDCap), which will be completed and electronically signed prior to participation (online supplemental file 5). After consent is provided, school staff will be contacted to schedule the data collection (ie, surveys, text messages and postimplementation focus groups). If ‘no’ is selected, participants will still be able to access and implement *TransformUs Secondary* in their schools.

### Sample size and power calculation

In 2023, there were 1453 secondary schools (Government and Independent schools) across Australia.^29^ *TransformUs* Primary (2018–23), reached 25% of primary schools over 4 years^23^; however, because of the shorter timeframe for this trial, we are expecting that no more than 5% of eligible schools will register for the programme via the *TransformUs Secondary* website, which equates to approximately ~70 schools. Based on response rates from the *TransformUs* Primary trial, we expect that no more than 40% of school staff will consent to take part in the research component. Making the intervention available to all schools enhances the generalisation of results by reaching diverse student populations, ensuring consistent implementation, and testing in varied real-world contexts (across school types and states and territories). This approach increases the sample size, allows for replication of outcomes and provides valuable feedback for continuous improvement.

### Measures

Implementation outcomes will be based on principles and recommendations in the PRACTical planning for Implementation and Scale-up (PRACTIS) Guide,^20^ key outcomes of the RE-AIM framework (reach, effectiveness, adoption, implementation and maintenance)^30^ (see online supplemental file 3), and implementation determinants (eg, context, acceptability, adaptability, feasibility and dose).^31^ The PRACTIS Guide provides an iterative step-by-step process to prospectively plan for implementation and scale-up of interventions in community settings, with the aim to enhance the potential translatability and impact of interventions in real-world settings. Data will be collected via (a) *TransformUs Secondary* registration data; (b) website analytics (eg, the number of overall page views per school and the number of users visiting the resource pages); (c) the Australian government My School website https://www.myschool.edu.au/ (eg, school type), (d) a brief online survey completed by consenting teachers (at baseline and follow-up) via REDcap; (e) text messages sent regularly to teachers will be used to assess the frequency of intervention delivery via text messages up to three times per term and (f) a focus group with teachers asking about their experiences delivering the programme, including any barriers or enablers.

At the end of the 6-month intervention period, a subsample of consenting teachers will be invited to participate in a focus group approximately 1-hour long. We will conduct up to six focus groups in up to three states/territories (eg, up to two focus groups per state/territory). Each focus group will include up to 12 teachers, ideally from the same school (teachers from no more than two schools will be included in one focus group). To ensure timely participation, teachers will receive SMS/email reminders prompting them to complete the survey and respond to the text messages. Online supplemental file 3 provides a description of the different quantitative and qualitative methods used to assess four of the RE-AIM dimensions (effectiveness outcomes will be assessed in the effectiveness trial only). Online supplemental file 4 provides a detailed description of the different methods used to assess implementation and effectiveness trial outcomes (ie, data source, relevant trial/s, targeted participants, time points and context), which are briefly described below.

### Teacher-level outcomes

#### Self-reported sociodemographic characteristics, physical activity and perceptions of school readiness, climate and environment

During the TransformUs website registration process, teachers will be asked to report their age range, gender and years of experience working in the education sector (online supplemental file 4A). All consenting teachers to the research component will be provided with a link to an online survey (REDcap) and will be asked to report, current teaching role (eg, year level/s, subjects), and reason for deciding to participate. In addition, teachers will *self-report their usual physical activity* level in the past week (ie, adherence to the Australian physical activity guidelines)^32^ (online supplemental file 4D). *School readiness*, *school climate*, *school* environment and policies and *current practice* will be assessed via the teacher survey. These items were adapted from the *TransformUs* primary school implementation trial^23^ and consist of 5-point Likert scales ranging from 1 (strongly disagree) to 5 (strongly agree): *teachers’ perceptions about school readiness* (eg, ‘People who work in this school are committed to implementation of *TransformUs Secondary’*); *school climate* (eg, ‘In my school teachers will be expected to use *TransformUs* strategies and will be supported to integrate *TransformUs* into existing teaching and/or school practices and policies’); *school environment/policies* (eg, ‘Physical activity is a top priority at our school’, ‘Our school has policies that promote physical activity’) and *current practice* (eg, ‘Currently my school has organised physical activities at lunch/recess that students are interested in’). In addition, *teachers’ level of competence* for certain aspects of active pedagogy (lesson planning, managing the class, managing curriculum demands) will be assessed (eg, ‘For each aspect of active teaching and movement integration, indicate how you perceive your level of competence’) using a 5-point Likert scale ranging from 1 (very incompetent) to 5 (very competent). *Teachers’ barriers* to implementation of active breaks and lessons in their classes (eg, insufficient training, lack of time) will be assessed on a 5-point Likert scale ranging from 1 (no barrier or does not inhibit) to 5 (a major barrier)/strongly inhibits) (online supplemental file 4D).

#### Intervention implementation

Teachers will report via online survey at the end of the intervention period, the frequency, duration and level of confidence for incorporating each of the components of the *TransformUs Secondary* intervention (table 1). Their commitment to continue to deliver these strategies in the future will also be assessed. Strategies that helped teachers implement *TransformUs Secondary* (eg, participating in the professional learning session, having a *TransformUs* champion, actively involving students) will be assessed based on 12 questions adapted from the TransformUs Primary survey and School Implementation Strategies, Translating ERIC Resources (SISTER) implementation strategies^33^ (online supplemental file 4D). In addition to the implementation survey, teachers’ frequency of intervention delivery will be assessed three times per term throughout the 6-month intervention period using text messages during and after school hours at different times. Teachers will receive regular messages on their mobile phones asking them to nominate by a corresponding number which of the strategies they implemented that week (eg, Which of the following TransformUs strategies have you implemented in the last week?) (online supplemental file 4H).

#### Teachers’ perceptions of the program reach, effectiveness, adoption, implementation and maintenance

*Teachers' perceptions of the programme reach, effectiveness, adoption, implementation and maintenance will be assessed via the teacher focus groups*. The qualitative questions have been framed around the implementation outcomes framework proposed by Proctor *et al* (2011)^34^ (online supplemental 4I).

### Data analysis

All statistical analyses will be performed using STATA 18 for Windows (StataCorp LP). Descriptive statistics will be used to summarise quantitative data collected from surveys (eg, teachers’ self-reported sociodemographic information, physical activity levels, perceptions of school readiness, climate and environment, as well as teacher practices, competence levels and perceived implementation barriers). The survey data regarding programme Reach, Adoption, Implementation and Maintenance will be also presented descriptively.

Similar descriptive statistics will be used to summarise the data for intervention implementation, including frequency, duration and confidence levels for incorporating active lessons, breaks and homework, as well as a commitment to continue implementing these strategies. Additionally, analyses will be conducted to examine associations between implementation strategies and outcomes, using regression analysis.

Linear or logistic regression models will be used to examine associations between various factors (such as teachers’ sociodemographic characteristics, physical activity levels, perceptions of school climate, etc) and implementation level, adjusting for multiple comparisons. The implementation levels will be determined using methods from a prior evaluation of the *TransformUs* efficacy trial.^35^ In brief, teachers will be categorised based on the extent of implementation, determined by the proportion of the entire intervention delivered, considering both dose-delivered and fidelity. The implementation levels will be classified as: (1) ‘low’ (<33% of the entire intervention delivered); (2) ‘moderate’ (33%–67% delivered) and (3) ‘high’ (>67% delivered).

Qualitative data will be transcribed and thematically analysed using NVivo V.14. The coding and theme development process will initially follow a deductive approach, guided by the study aims and domains within Proctor’s implementation outcomes framework.^34^ Subsequently, an inductive method will be employed, driven by the content of the data itself. Two independent researchers will be responsible for coding the data.

### Effectiveness trial

The effectiveness trial will employ a pragmatic waitlist control design. Secondary schools will be assigned (based on the school availability and timing requirements) to the *TransformUs Secondary* intervention (n=5 schools) or a waitlist control ‘usual practice’ condition (n=5 schools). Waitlist control schools will receive the intervention after 6 months (approximately 2–3 school terms).

### Participants and recruitment

The effectiveness trial will be conducted in a sample of Victorian secondary schools. Schools that meet the following criteria will be eligible to participate in the study: (1) Government, Independent and Catholic secondary schools; (2) have >200 students in Years 7–10 and (3) not fully elective/sports/performing arts/agriculture/boarding schools. Participants in the effectiveness trial will include school leaders, teachers and students. Recruitment for the trial will commence in July 2023 and continue until recruitment numbers have been reached with a deadline of December 2024. As school recruitment is happening over an 18-month period, school study commencement will be balanced in pairs to reduce seasonality effects. Therefore, baseline data collection at each school will not take place until at least two schools are recruited (one intervention and one control).

Recruitment of eligible schools will occur via direct email contact by the research team, by communications sent from key education organisation partners (ie, education departments, the Australian Council for Health, Physical Education and Recreation) and by presentations at teacher/education conferences. Targeted school recruitment will be used to ensure recruiting a range of schools from urban, regional and rural settings, socioeconomic areas and of different types (ie, Government, Catholic and Independent schools). The Index of Community Socio-educational Advantage (ICSEA) will serve as the socioeducation advantage variable.^36^ This index includes information regarding parental education and occupation, geographical location and Indigenous student representation.^37^ Therefore, the targeted school recruitment will aim to categorise the schools from a higher (ICSEA ≤1000) and a lower (ICSEA >1000) socioeducation advantage stratum and by school type. This process will produce four strata that represent the distribution of Victorian schools:^29^ (a) Government high ICSEA, (b) Government low ICSEA, (c) Independent high ICSEA and (d) Catholic high ICSEA, prioritising Government low ICSEA. Schools that express interest (eg, via email, or via an expression of interest quick response (QR) code linked to Qualtrics) will be sent the plain language statement and consent form and an organisational consent form on Qualtrics or REDCap.

Following school consent, *TransformUs Secondary* information will be sent to school staff (school leaders and Year 7–10 teachers) via the school’s regular channels of communication (eg, intranet, email or other methods requested by the school). Once organisational and teacher consent is received, plain language statement and consent form documentation will be sent to students (ie, students attending lessons delivered by consenting teachers) and parents (via the school’s regular channels of communication) (online supplemental file 5). There will be two tiers of consent for students. Parents (on behalf of their child) and adolescents will be invited to consent to the following assessments: accelerometer, surveys and classroom observations. In addition, one optional assessment (posture device) will be an opt-in that adolescents can choose to take part in.

### Blinding

Due to the inability to blind schools and teachers to the programme strategies, the study will be conducted as an open trial. Blinding cannot be assured as participation may be disclosed by school staff or students or evident in the school setting (eg, *TransformUs* signage, active environments). The statistician undertaking data analysis will be blinded to the study group, with unlabelled numeric treatment codes being provided.

### Sample size and power calculations

Students will be recruited from 4-year-levels (Years 7, 8, 9 and 10) from each of the 10 schools. It is estimated an average of 15 students will be recruited per year level, enabling at least 12 for analysis, assuming up to 20% dropout. We will conservatively apply a design effect based on an intraclass correlation coefficient (ICC) of 0.03. Assuming an SD for the MVPA outcome of 17 min/day (Corder *et al* 2020), we will have 80% power to detect a minimum difference between groups of 7 min/day MVPA, for α=0.05. Therefore, participants in the effectiveness trial will include one nominated school leader per school (n=up to 10); approximately eight Year 7–10 teachers per school (n=up to 80) and all Year 7–10 secondary students attending classes with the consenting teachers (only consenting student will take part in the evaluation). The recruitment target will be approximately 600 students in total (approximately n=60 students per school). Sample size calculations were conducted in Stata/SE 16 using the power two means command.

Including an effectiveness trial that is accessible to all schools in Victoria enhances the generalisability of the results by incorporating diverse student populations representing various educational contexts, needs and resources. This broad approach improves the reliability of findings, reflecting how the intervention performs across different demographics and settings, making the results more applicable to other schools in the state. However, using a subsample may limit generalisation as it may not fully represent the student population’s diversity. Smaller or more localised samples could be influenced by specific local conditions, reducing the ability to make broader conclusions. While subsamples offer valuable insights, they may lack the comprehensiveness needed for strong generalisations, which we have acknowledged as a limitation.

### Intervention group

After baseline data collection, teachers in the intervention group will attend a 1-hour face-to-face professional learning session. This session will cover the following topics: defining and understanding the benefits of physical activity, defining and understanding the impact of sedentary behaviour, information about *TransformUs Secondary* and explaining the six programme components, practical demonstrations of how to plan for and deliver active breaks and active lessons in their teaching and how to navigate and obtain the programme resources from the website, with opportunities to experience and deliver active breaks and lessons in an environment that fosters meaningful activity. Teachers will be encouraged to register on the *TransformUs* Website, plan for and use the programme resources in all their lessons, encourage their students to participate as champions/leaders for activities inside and outside the classroom, visit the website regularly and request assistance from the research team when needed via *TransformUs Secondary*’s email or phone. In addition, schools will receive $A2000 to assist with programme implementation. Teachers and school leaders will be asked to decide how to use the funds to support the delivery of prioritised strategies (eg, active equipment, line markings, height adjustable desks and classroom signage).

### Waitlist control group

Schools assigned to the waitlist control group will be asked to continue their usual lesson delivery and will receive professional learning, access to the online programme resources and $A2000 implementation funds at the completion of the postintervention data collection.

### Measures

All assessments will occur at two time points. Baseline assessments will be conducted before the beginning of the trial and repeated after 6 months or 2–3 school terms of intervention delivery. The measures will be taken by trained research staff. Student-level outcomes will be used to determine the effectiveness of the programme on adolescents’ physical activity and sedentary time (primary outcomes) and on sleep duration and quality, class engagement, well-being, students’ physical activity during morning recess and lunch breaks, on-task behaviour in class and sitting time (secondary outcomes). Students with informed consent will complete a brief online survey, approximately 10–15 min in duration, at school. Following the survey, the research team will distribute and provide instructions for the use of the activity device to students with consent in a room assigned by the school. Meanwhile, teachers will receive a survey link via their work email, allowing them to complete the assessment at their convenience. To ensure timely participation, students will receive SMS reminders to wear the devices, and teacher will receive a reminder to complete the survey.

### Student-level outcomes

#### Device-assessed physical activity, sedentary time and sleep

Participants will wear an ActiGraph GT9X accelerometer (ActiGraph LLC, Pensacola, Florida, USA) on their non-dominant wrist for 24 hours a day for eight consecutive days at both measurement points. This device measures the acceleration and deceleration of human movement. Acceleration data can be used to identify the intensity of a given movement as well as its underlying temporal pattern (ie, frequency, duration) over defined epoch durations (eg, during recess and lunch periods).^38^ This device has demonstrated validity and reliability in adolescents.^39^ Data will be reduced using evidenced-based, best-practice procedures at the time of analysis (eg, open-source package General Goodness of Fit (GGIR) in R software) to obtain the time (during school, recess and lunch, and waking hours) spent sedentary and in MVPA on an average school day and average weekday and weekend days (primary outcomes) and light, moderate and vigorous physical activity (during school, recess and lunch, average school day and average day (weekday and weekends)) and average sleep duration (secondary outcomes).

#### Device-assessed sitting time

Students can also opt to wear an activPAL3 inclinometer (PAL Technologies, Glasgow, UK) on the front of their right thigh (at the mid-point), attached via an elastic belt during waking hours, for 8 days at each of the measurement points. This device (sampling at 10 Hz) detects limb position and is a valid and reliable device for measuring sitting, standing and stepping time in children and adolescents.^40^ Average minutes (during school, recess and lunch, average school day and average day (weekday and weekend days)), spent sitting, in continuous sitting bouts, standing, stepping and the number of transitions from sitting will be computed from activPAL data using ProcessingPAL.^41^

#### Self-reported sociodemographic characteristics, physical activity, sleep, wellbeing and class engagement

Students will self-report their *sociodemographic characteristics*, including age, gender, year level, country in which they were born and main language spoken at home. The Youth Activity Profile will be self-administered to capture *school-related physical activity and sedentary behaviour*. This 7-day recall instrument is designed to facilitate physical activity assessment in youth and has been validated for school-based assessments.^42^ The 15-item questionnaire comprised three sections (school day, out-of-school and sedentary behaviour), with five questions per section. During context-specific time segments, participants are asked to recall their physical activity and sedentary behaviour over the past 7 days^42^ (online supplemental file 4E). The *Adolescent Sleep Wake Scale* (ASWS) is a brief questionnaire, consisting of 20 items, that has been validated to assess overall sleep quality and insomnia symptoms in adolescents (ages 12–18).^43^ The ASWS includes five subscales that measure different aspects of sleep behaviour, including Going to Bed, Falling Asleep, Maintaining Sleep, Reinitiating Sleep and Return to Wakefulness. Scores for the total scale and each subscale are calculated as the average of the respondent’s answers to all items, rated on a scale from 1 (‘Always’) to 6 (‘Never’). Higher scores on the ASWS indicate better sleep quality. In addition, several questions from the self-report Child and Adolescent Sleep Checklist (CASC) will be used to measure sleep duration.^44^ This included questions on total sleep time, bedtime and wake time on weekdays and weekends over the past week. The *Engagement, Perseverance, Optimism, Connectedness, and Happiness* questionnaire is designed to assess positive psychological functioning and emotions in adolescents and encompasses five positive psychological traits: engagement, perseverance, optimism, connectedness and happiness.^45^ Students will be asked to rate 10 items on a 5-point scale, ranging from ‘almost never’ to ‘almost always’ and 10 items on a scale ranging from ‘not at all like me’ to very much like me’. Scores are then calculated for each of the five domains, providing an indication of an individual’s level of positive psychological functioning and feelings.^45^ Higher scores indicate more engagement, perseverance, optimism, connectedness, and happiness (online supplemental file 4E). The behavioural and emotional/motivational aspects of students’ *classroom engagement* will be captured using the Engagement vs Disaffection with Learning survey.^46^ Respondents use a 4-point Likert-type scale ranging from 1 (not at all true) to 4 (very true). The tool consists of four dimensions and a total of 20 items hereafter organised to assess behavioural engagement, behavioural disaffection, emotional engagement and emotional disaffection at school. Higher scores on each dimension indicate more engagement or disaffection (online supplemental file 4E).

#### Students’ on-task behaviour

*Students’ on-task behaviour* will be assessed with direct observations, using momentary sampling with 15-s observation intervals. This approach is aligned with what was adopted in previous research^47^ and involves at least one researcher to be in the classroom and routinely observing students (1:6 observer/student ratio) during a classroom lesson. The observer will look at a target consented student for 5 s and use the remaining 10 s to record the momentary-observed behaviour and some contextual information (ie, the student’s movement behaviour and information on the task being conducted at the time). The same target students will be observed for four intervals (ie, 1 min) before moving to the next student. In a 30-min observation session, each student will be observed 20 times. Observed students’ behaviours will be classified as follows: focused on-task (the student is focused on the task assigned by the teacher, eg, reading, writing, asking/answering questions, active in a purposeful way that aligns with learning intentions), passive off-task (the student is not focusing on the assigned task and shows passive disengagement, eg, not focused, daydreaming, staring, drinking water), disruptive off-task (eg, the student is not focusing on the assigned task and shows a disruptive behaviour; not focused walking around, playing with irrelevant objects, fidgeting), absent (eg, the student left the classroom). Contextual information relating to students’ movement behaviours will be classified as follows: sitting, standing, walking slow, walking fast and moving fast (eg, jumping). Information on the setting/task will be coded as follows: independent task, paired task, group task, teacher-led activity, test and other. To ensure consistency in the observation and data collection processes, an online observation tool to record students’ behaviour was developed in the Qualtrics survey by the research team (online supplemental file 4F).

#### Student attendance

Overall student attendance data will be collected for each participating school at baseline and 6 months. The percentage of actual full-time equivalent student days attended as a percentage of the total number of possible student days attended over the (reporting) period will be collected from the Australian Curriculum, Assessment and Reporting Authority website (https://www.acara.edu.au/contact-us/acara-data-access).

#### Observed physical activity during recess and lunch

Students’ physical activity during recess and lunch will be assessed using the System for Observing Play and Leisure Activity in Youth (SOPLAY) at baseline and follow-up. SOPLAY is a validated tool for directly observing physical activity and associated environmental characteristics in free-play settings.^48^ SOPLAY provides objective data on the number of participants and their physical activity levels during play and leisure opportunities in targeted areas. The research staff will define the target areas. Separate scans are made for males and females, and simultaneous entries for contextual characteristics of areas including their accessibility, usability, and whether supervision, organised activities and equipment are provided. A map of the zones and SOPLAY data collection sheets will be provided to each research assistant. Observations will begin 5 min into recess and 10 min into lunch and occur every 5 min. Research assistants will rotate clockwise around the zones, with each group starting in a different zone. Observations will end 5 min before the end of recess/lunch. All observations will be recorded on the SOPLAY data collection sheet. The predominant type of activity engaged in by area users will also be recorded (eg, basketball, dance) (online supplemental file 4G).

#### Engagement with the intervention strategies

Students’ experience/access to intervention strategies will be assessed with seven survey questions developed by the research team. The questions will determine if their school has organised (eg, by teachers/schools or students) physical activities at lunch/recess, outdoor and classroom environment that promotes movement, teachers who deliver active lessons/breaks and teachers who give active homework. Respondents use a 6-point Likert-type scale ranging from 1 (doesn't have) to 6 (strongly agree) (online supplemental file 4E).

### Teacher-level outcomes

Self-reported sociodemographic data, physical activity and perceptions of school readiness, climate and environment; teacher practices, competence levels and implementation barriers and teachers’ perceptions of the programme reach, effectiveness, adoption, implementation and maintenance will be assessed using the same survey described for the implementation trial section and detailed in the online supplemental file 4D).

### School-level outcomes

#### School grounds audit

The ‘Artificial Intelligence-based School Audit’ (AISA) online audit tool, recently developed by the research team, will be used to assess the school’s physical environment, to be assessed as a potential moderator of the intervention effects. The audit tool quantifies the dimension (m^2^) of the school footprint and the percentage of the school grounds taken up by buildings and on-site car parking, sports courts, green spaces (eg, trees, open green spaces), playgrounds and covered areas (eg, shade sails). AISA uses an advanced artificial intelligence methodology and deep learning segmentation pipeline to quickly and accurately label the above environmental features using date-stamped satellite images of school grounds. Some additional environmental characteristics not captured by AISA will be audited in person (eg, sports or playground equipment, playground/sports courts markings, playgrounds/outdoor spaces separated by a physical barrier, aesthetics of facilities, bicycle parking).

### Data analysis

Analysis of the primary and secondary outcomes will be performed using Stata 18 for Windows (StataCorp LP) and RStudio V.1.4.453 (R V.3.6.3; R Foundation for Statistical Computing, Vienna, Austria).

Descriptive analyses will be employed to depict the quantitative data collected for all variables of interest, including students’ demographic characteristics, physical activity, sedentary behaviour, sleep, well-being, class engagement, school environment and intervention implementation measures (eg, school audit data) at each time point. Data will be examined for missing values, outliers and distributional assumptions. Teachers’ demographic characteristics and survey responses from both intervention and control groups will be described.

For the primary outcomes, random intercept linear mixed models will be used (where the unit of the analysis (students) will be nested within year levels within schools) to examine the intervention effects on primary (physical activity and sedentary time) and secondary (sleep and well-being, class and school engagement, reducing sitting time, increasing light, moderate and vigorous physical activity, self-report physical activity/sedentary behaviours, observed physical activity during recess and lunch time and engagement with the intervention strategies) outcomes among adolescents. All models will be adjusted for measured confounders age, gender, baseline values, socioeducational advantage strata (high vs low ICSEA) and school type (Government, Independent, Catholic).

The programme’s reach, adoption, implementation, and organisational-level maintenance aspects will be presented descriptively. Linear or logistic regression models will be used to compare differences between intervention and control groups in teacher perceptions of school readiness, climate, and environment, as well as teacher practices, competence levels, and implementation barriers, adjusting for baseline values as covariates. The school grounds audit data will be analysed using descriptive statistics to characterise the physical environment of intervention and control schools and interactions with the intervention effects will be examined to determine if there was a potential moderator effect by school environment.

Qualitative data will be transcribed and thematically analysed using NVivo V.14. The coding and theme development process will initially follow a deductive approach, guided by the study aims and domains within the Proctor’s implementation outcomes framework.^34^ Subsequently, an inductive method will be employed, driven by the content of the data itself. Two independent researchers will be responsible for coding the data.

## Ethics and dissemination

These trials were approved by the Deakin University Human Research Ethics Committee (2021–269) and by following education authorities: Australian Capital Territory Education Directorate (RES 2317), New South Wales Department of Education (2022253), Northern Territory Department of Education (20865), Victoria Department of Education (2023_004712), Queensland Department of Education (550/27/2585), South Australian Department of Education (2022–0019), Tasmanian Department of Education (2022–25), Western Australian Department of Education (D23/1152724) and Melbourne Archdiocese Catholic Schools (1232). Before participating in surveys or interviews/focus groups, partners, schools/teachers and students will need to provide informed and signed consent. Additionally, parents will be required to give consent for their child’s involvement in assessments as a component of the effectiveness trial. All supporting documentation will stress the voluntary nature of participation and clearly state that there will be no adverse consequences if participants choose not to take part in the research. Throughout the project, exclusive access to participants’ data will be granted solely to the research team. All computers and files will be safeguarded with password protection. Results from this study will be disseminated through peer-reviewed journal articles, scientific conferences, summary reports to students and schools and meetings with key organisations.

## supplementary material

10.1136/bmjopen-2024-090468 online supplemental file 1
