# Supplementary material for: Protocol of the TransformUs Secondary schools program: a type II hybrid implementation-effectiveness trial to increase adolescents’ physical activity and reduce sedentary time in secondary schools
Source: BMJ Open. 2025 Feb 10;15(2):e090468. doi: 10.1136/bmjopen-2024-090468 (PMC11815412; doi:10.1136/bmjopen-2024-090468)
Supplement: online supplemental file 1 [file bmjopen-15-2-s001.docx]

**Online Supplementary file 1: Timeline of *TransformUs Secondary***

|  | 2021 | | | | 2022 | | | | 2023 | | | | 2024 | | | |
| --- | --- | --- | --- | --- | --- | --- | --- | --- | --- | --- | --- | --- | --- | --- | --- | --- |
| **Term** | **1** | **2** | **3** | **4** | **1** | **2** | **3** | **4** | **1** | **2** | **3** | **4** | **1** | **2** | **3** | **4** |
| **Preparation activities** |  |  |  |  |  |  |  |  |  |  |  |  |  |  |  |  |
| Interviews with academic and key organisations |  |  |  |  |  |  |  |  |  |  |  |  |  |  |  |  |
| Co design workshops (students, teachers, school leaders) |  |  |  |  |  |  |  |  |  |  |  |  |  |  |  |  |
| TransformUs Pilot (3 Victorian Schools) |  |  |  |  |  |  |  |  |  |  |  |  |  |  |  |  |
| Co-design workshops (stakeholders WA, SA, QLD) |  |  |  |  |  |  |  |  |  |  |  |  |  |  |  |  |
| Resource development |  |  |  |  |  |  |  |  |  |  |  |  |  |  |  |  |
| **Key organisations' engagement - Dissemination** |  |  |  |  |  |  |  |  |  |  |  |  |  |  |  |  |
| Email existing and potential key organisations (WA, SA, QLD, NT,ACT) |  |  |  |  |  |  |  |  |  |  |  |  |  |  |  |  |
| Stakeholder meetings |  |  |  |  |  |  |  |  |  |  |  |  |  |  |  |  |
| Send stakeholder toolkit |  |  |  |  |  |  |  |  |  |  |  |  |  |  |  |  |
| TransformUs Secondary - website launch per state |  |  |  |  |  |  |  |  |  |  |  |  |  |  |  |  |
| **Effectiveness trial** |  |  |  |  |  |  |  |  |  |  |  |  |  |  |  |  |
| Launch |  |  |  |  |  |  |  |  |  |  |  |  |  |  |  |  |
| Wave 1 (4 schools) |  |  |  |  |  |  |  |  |  |  |  |  |  |  |  |  |
| Baseline measures |  |  |  |  |  |  |  |  |  |  |  |  |  |  |  |  |
| Intervention implementation* |  |  |  |  |  |  |  |  |  |  |  |  |  |  |  |  |
| Post-intervention measures |  |  |  |  |  |  |  |  |  |  |  |  |  |  |  |  |
| Wave 2 (6 schools) |  |  |  |  |  |  |  |  |  |  |  |  |  |  |  |  |
| Baseline measures |  |  |  |  |  |  |  |  |  |  |  |  |  |  |  |  |
| Intervention implementation* |  |  |  |  |  |  |  |  |  |  |  |  |  |  |  |  |
| Post-intervention measures |  |  |  |  |  |  |  |  |  |  |  |  |  |  |  |  |
| **Implementation trial** |  |  |  |  |  |  |  |  |  |  |  |  |  |  |  |  |
| Launch |  |  |  |  |  |  |  |  |  |  |  |  |  |  |  |  |
| Recruitment |  |  |  |  |  |  |  |  |  |  |  |  |  |  |  |  |
| Baseline data collection |  |  |  |  |  |  |  |  |  |  |  |  |  |  |  |  |
| T2 data collection |  |  |  |  |  |  |  |  |  |  |  |  |  |  |  |  |

**Legend: (***)intervention schools only; waist-list control schools will received the intervention after post-intervention measures

Online Supplementary file 2

SPIRIT 2013 Checklist: Recommended items to address in a clinical trial protocol and related documents*

| **Section/item** | **ItemNo** | **Description** | **Location in manuscript** |
| --- | --- | --- | --- |
| **Administrative information** | | |  |
| Title | 1 | Descriptive title identifying the study design, population, interventions, and, if applicable, trial acronym | 1 |
| Trial registration | 2a | Trial identifier and registry name. If not yet registered, name of intended registry | 4 |
|  | 2b | All items from the World Health Organization Trial Registration Data Set | N/A |
| Protocol version | 3 | Date and version identifier | 1 |
| Funding | 4 | Sources and types of financial, material, and other support | 22 |
| Roles and responsibilities | 5a | Names, affiliations, and roles of protocol contributors | 1 & 22 |
|  | 5b | Name and contact information for the trial sponsor | 22 |
|  | 5c | Role of study sponsor and funders, if any, in study design; collection, management, analysis, and interpretation of data; writing of the report; and the decision to submit the report for publication, including whether they will have ultimate authority over any of these activities | 22 |
|  | 5d | Composition, roles, and responsibilities of the coordinating centre, steering committee, endpoint adjudication committee, data management team, and other individuals or groups overseeing the trial, if applicable (see Item 21a for data monitoring committee) | N/A |
| **Introduction** |  |  |  |
| Background and rationale | 6a | Description of research question and justification for undertaking the trial, including summary of relevant studies (published and unpublished) examining benefits and harms for each intervention | 4-5 |
|  | 6b | Explanation for choice of comparators | 5 |
| Objectives | 7 | Specific objectives or hypotheses | 5 |
| Trial design | 8 | Description of trial design including type of trial (eg, parallel group, crossover, factorial, single group), allocation ratio, and framework (eg, superiority, equivalence, noninferiority, exploratory) | 6 |
| **Methods: Participants, interventions, and outcomes** | | |  |
| Study setting | 9 | Description of study settings (eg, community clinic, academic hospital) and list of countries where data will be collected. Reference to where list of study sites can be obtained | 9 & 17 |
| Eligibility criteria | 10 | Inclusion and exclusion criteria for participants. If applicable, eligibility criteria for study centres and individuals who will perform the interventions (eg, surgeons, psychotherapists) | 9 |
| Interventions | 11a | Interventions for each group with sufficient detail to allow replication, including how and when they will be administered | 6-7 |
|  | 11b | Criteria for discontinuing or modifying allocated interventions for a given trial participant (eg, drug dose change in response to harms, participant request, or improving/worsening disease) | N/A |
|  | 11c | Strategies to improve adherence to intervention protocols, and any procedures for monitoring adherence (eg, drug tablet return, laboratory tests) | 7 |
|  | 11d | Relevant concomitant care and interventions that are permitted or prohibited during the trial | 11 |
| Outcomes | 12 | Primary, secondary, and other outcomes, including the specific measurement variable (eg, systolic blood pressure), analysis metric (eg, change from baseline, final value, time to event), method of aggregation (eg, median, proportion), and time point for each outcome. Explanation of the clinical relevance of chosen efficacy and harm outcomes is strongly recommended | 11-16, 18 |
| Participant timeline | 13 | Time schedule of enrolment, interventions (including any run-ins and washouts), assessments, and visits for participants. A schematic diagram is highly recommended (see Figure) | Appendix A |
| Sample size | 14 | Estimated number of participants needed to achieve study objectives and how it was determined, including clinical and statistical assumptions supporting any sample size calculations | 10 & 17 |
| Recruitment | 15 | Strategies for achieving adequate participant enrolment to reach target sample size | 9-10 & 17 |
| **Methods: Assignment of interventions (for controlled trials)** | | |  |
| Allocation: |  |  |  |
| Sequence generation | 16a | Method of generating the allocation sequence (eg, computer-generated random numbers), and list of any factors for stratification. To reduce predictability of a random sequence, details of any planned restriction (eg, blocking) should be provided in a separate document that is unavailable to those who enrol participants or assign interventions | 9 |
| Allocation concealment mechanism | 16b | Mechanism of implementing the allocation sequence (eg, central telephone; sequentially numbered, opaque, sealed envelopes), describing any steps to conceal the sequence until interventions are assigned | 9 |
| Implementation | 16c | Who will generate the allocation sequence, who will enrol participants, and who will assign participants to interventions | 9 |
| Blinding (masking) | 17a | Who will be blinded after assignment to interventions (eg, trial participants, care providers, outcome assessors, data analysts), and how | 10 |
|  | 17b | If blinded, circumstances under which unblinding is permissible, and procedure for revealing a participant's allocated intervention during the trial | 10 |
| **Methods: Data collection, management, and analysis** | | |  |
| Data collection methods | 18a | Plans for assessment and collection of outcome, baseline, and other trial data, including any related processes to promote data quality (eg, duplicate measurements, training of assessors) and a description of study instruments (eg, questionnaires, laboratory tests) along with their reliability and validity, if known. Reference to where data collection forms can be found, if not in the protocol | 11 |
|  | 18b | Plans to promote participant retention and complete follow-up, including list of any outcome data to be collected for participants who discontinue or deviate from intervention protocols | 11-12 &18 |
| Data management | 19 | Plans for data entry, coding, security, and storage, including any related processes to promote data quality (eg, double data entry; range checks for data values). Reference to where details of data management procedures can be found, if not in the protocol | 16 & 21 |
| Statistical methods | 20a | Statistical methods for analyzing primary and secondary outcomes. Reference to where other details of the statistical analysis plan can be found, if not in the protocol | 16 & 21 |
|  | 20b | Methods for any additional analyses (eg, subgroup and adjusted analyses) | 16 & 21 |
|  | 20c | Definition of analysis population relating to protocol non-adherence (eg, as randomized analysis), and any statistical methods to handle missing data (eg, multiple imputation) | 16 & 21 |
| **Methods: Monitoring** | | |  |
| Data monitoring | 21a | Composition of data monitoring committee (DMC); summary of its role and reporting structure; statement of whether it is independent from the sponsor and competing interests; and reference to where further details about its charter can be found, if not in the protocol. Alternatively, an explanation of why a DMC is not needed | N/A |
|  | 21b | Description of any interim analyses and stopping guidelines, including who will have access to these interim results and make the final decision to terminate the trial | N/A |
| Harms | 22 | Plans for collecting, assessing, reporting, and managing solicited and spontaneously reported adverse events and other unintended effects of trial interventions or trial conduct | N/A |
| Auditing | 23 | Frequency and procedures for auditing trial conduct, if any, and whether the process will be independent from investigators and the sponsor | N/A |
| **Ethics and dissemination** | | |  |
| Research ethics approval | 24 | Plans for seeking research ethics committee/institutional review board (REC/IRB) approval | 21 |
| Protocol amendments | 25 | Plans for communicating important protocol modifications (eg, changes to eligibility criteria, outcomes, analyses) to relevant parties (eg, investigators, REC/IRBs, trial participants, trial registries, journals, regulators) | N/A |
| Consent or assent | 26a | Who will obtain informed consent or assent from potential trial participants or authorized surrogates, and how (see Item 32) | 10, 11, 16, 17 & 21 |
|  | 26b | Additional consent provisions for collection and use of participant data and biological specimens in ancillary studies, if applicable | N/A |
| Confidentiality | 27 | How personal information about potential and enrolled participants will be collected, shared, and maintained in order to protect confidentiality before, during, and after the trial | 21 |
| Declaration of interests | 28 | Financial and other competing interests for principal investigators for the overall trial and each study site | 22 |
| Access to data | 29 | Statement of who will have access to the final trial dataset, and disclosure of contractual agreements that limit such access for investigators | 21 |
| Ancillary and post-trial care | 30 | Provisions, if any, for ancillary and post-trial care, and for compensation to those who suffer harm from trial participation | N/A |
| Dissemination policy | 31a | Plans for investigators and sponsor to communicate trial results to participants, healthcare professionals, the public, and other relevant groups (eg, via publication, reporting in results databases, or other data sharing arrangements), including any publication restrictions | 21 |
|  | 31b | Authorship eligibility guidelines and any intended use of professional writers | N/A |
|  | 31c | Plans, if any, for granting public access to the full protocol, participant-level dataset, and statistical code | N/A |
| **Appendices** |  |  |  |
| Informed consent materials | 32 | Model consent form and other related documentation given to participants and authorized surrogates | N/A |
| Biological specimens | 33 | Plans for collection, laboratory evaluation, and storage of biological specimens for genetic or molecular analysis in the current trial and for future use in ancillary studies, if applicable | N/A |

*It is strongly recommended that this checklist be read in conjunction with the SPIRIT 2013 Explanation & Elaboration for important clarification on the items. Amendments to the protocol should be tracked and dated. The SPIRIT checklist is copyrighted by the SPIRIT Group under the Creative Commons "[Attribution-NonCommercial-NoDerivs 3.0 Unported](http://www.creativecommons.org/licenses/by-nc-nd/3.0/)" license.

**Online Supplementary File 3 RE-AIM operationalisation, data sources, evaluation data and participant groups**

| **RE-AIM framework** | **Operationalization** | **Data source/tool (participant groups)** | **Outcome data** |
| --- | --- | --- | --- |
| Reach | - Estimate the number of teachers and students who might have been exposed to *TransformUs Secondary* - Examine if participant schools are representative of the Australian schools in term of school size, SES and location | - *TransformUs* website using web analytics (teachers) - *TransformUs Secondary* registration data (teachers) - MySchool website, School student enrolment and characteristics data (schools, students) | - The number, proportion and representativeness of teachers and students - Students’ characteristics: gender distribution, SES, location and size, proportion if non-English speaking and indigenous students |
| Effectiveness (*) | Effectiveness trial data |  |  |
| Adoption | - Total number and representativeness of schools and teacher that participated in *TransformUs Secondary* - Proportion of teachers and leaders who complete the teacher professional learning session (online) | - *TransformUs* website using web analytics (teachers) - Online registration process (school and teachers) - Teachers’ online survey (teachers) - My school website (school and students) | - The number, proportion and representativeness of schools and teachers; and variation of adoption across settings and deliverers - School characteristics are school size, SES, location, proportion if non-English speaking and indigenous students - Teachers' characteristics: age, sex, years of teaching experience - Proportion of teachers who complete the teacher professional development session |
| Implementation | The extent to which the six components of *TransformUs Secondary* are delivered as intended | - *TransformU*s website using web analytics (teachers) - Teacher online survey (teachers) - Text messages (teachers) - Post-implementation teacher focus groups (teachers) | - The intervention teachers' fidelity to the intervention, including consistency of delivery as intended; adaptations made to interventions and implementation strategies - Dose and quality of delivery |
| Maintenance | The extent to which *TransformUs Secondary* will be institutionalised in schools | - Teacher online survey (teachers) - Post-implementation teacher focus groups (teachers) | - The extent to which the program has or could become institutionalized or part of routine practice; the long-term effects of the program on teaching practices and student outcomes after the intervention is completed |

**Online Supplementary File 4:** Detailed description of the different methods used to assess implementation and effectiveness of trial outcomes.

| **Data source/Tool** | **Content** (response options) |
| --- | --- |
| 1. ***TransformUs Secondary* registration data (*) – TransformUs website**   Trial:  IT & ET  Participants:  School Staff  Timepoint:  Baseline | 1. Where are you located (ACT, NSW, NT, QLD, SA, TAS, VIC, WA) 2. What best describes you? (Select as many as appropriate: Assistant principal, Casual relief teacher, Classroom teacher, County/district/regional coordinator, education department employee, Education support in a school, Health promotion officer, Lecturer, PE teacher, Pre-service teacher, Principal, School administrator, School wellbeing coordinator, Specialist teacher, Other (please specify) 3. What organisation do you work for? Open-ended response (For Health promotion officer) 4. Are you currently working in a school or university? (Yes – No) 5. Describe the workplace (Primary, Secondary, Special school) 6. School type (Catholic [Melbourne Archdiocese, Other], Government, Independent) 7. Do you have students with additional needs in your classroom (yes - No) 8. Search for the school you are working at (Dropdown box options) 9. Your name 10. Gender (Female, Male, other, prefer not to say) 11. Age range (18-20, 21-30, 31-40, 41-50, 51-60, +61) 12. How many years of experience working in education do you have? 13. Email and password 14. How did you hear about us? (Please indicate: Department of Education and Training Victoria VicHealth, Australian Council for Health, Physical Education and Recreation, Victoria; Victorian Principals Association; Independent Schools Victoria; Peak Phys Ed; Bluearth; Cancer Council Victoria/ School Achievement Program; Catholic Education Office; Municipal Association of Victoria; Victorian Curriculum and Assessment Authority; Department of Health and Human Services; Hawthorn FC; DPV Health; Kiddo; Geelong FC (Healthy Heroes); Teacher advocate within the school or Principal of your school; Other Principal/school doing TransformUs; Social Media; Other (please specify)) 15. Would you like to receive email updates relating to new resources and content uploaded to the TransformUs website to assist you with your active teaching? 16. We would like to invite you to take part in research to provide feedback that will inform the continual improvement of TransformUs. This is completely voluntary and would involve surveys and an interview. 17. Would you like to participate in the Secondary research? (Yes – No) |
| 1. **Website analytics**   Trial:  IT & ET  Participants:  Registered participants  Timepoint:  Evaluation period | 1. Number of overall page views per users 2. Number of users visiting the resource pages 3. Which program components are downloaded 4. Which aspect of the website are most and least accessed? 5. Proportion of teachers who complete the teacher professional development session |
| 1. **My school website**   Trial:  IT & ET  Participants:  Participants’ schools  Timepoint:  Baseline | 1. School size 2. School socio-economic status 3. School location 4. School student enrolment: proportion if non-English speaking and indigenous students |
| 1. **Online survey (via Redcap)**   Trial:  IT & ET  Participants:  Teacher - participants who agree to take part in the research component  Timepoint:  Baseline  Follow up | 1. Self-reported sociodemographic data:    1. How would you describe your role? (Assistant principal, Casual relief teacher, Education support in a school, Principal, School administrator, School wellbeing coordinator, Teacher, other [please specify])    2. Are you currently teaching any year levels in the school? (yes -no)    3. Which year levels? (Please select all that apply: year 7, 8, 9, 10,11, 12)    4. What subjects do you currently teach? (Art, drama, English, Health, Humanities, LOTE, Maths, Physical Education, Science, other [please specify])    5. How long have you been teaching in an Australian secondary school? (please specify)^(a)^    6. What is the name of the school you are currently employed at? (Please specify the campus as appropriate) 2. Physical activities in your free time:    1. In a typical week, how much time do you spend in total on moderate and vigorous physical activities where your heartbeat increases and you breathe faster (e.g., brisk walking, cycling as a means of transport or exercise, heavy gardening, running or recreational sports). Only include activities that lasted at least 10 minutes at a time. (Hours per week [please specify] and Minutes per week [please specify])    2. How much of the time that you spend on physical activities in a typical week, which you indicated above, do you spend in total on vigorous physical activities? This includes activities that get your heart racing, make you sweat and leave you so short of breath that speaking becomes difficult (e.g., swimming, running, cycling at high speeds, cardio training, weight lifting or team sports). (Hours per week [please specify] and Minutes per week [please specify])    3. In a typical week, on how many days do you do muscle-strengthening activities (e.g., push-ups, pull-ups, squats or lunges, lifting weights, household tasks that involve lifting, carrying or digging)? (please select: no day, 1 day, 2 days, 3 days, 4 days, 5 days, 6 days, 7 days) 3. Organisational readiness: Please indicate (Select strongly disagree, disagree, Neither agree nor disagree, Agree, Strongly agree) how much you agree or disagree with the following statements People who work in this school^(c)^:    1. Are committed to implementing TransformUs    2. Want to implement TransformUs    3. Are motivated to implement TransformUs    4. Feel confident that they can handle the challenges that might arise in implementing TransformUs    5. Feel confident that they can keep track of progress in implementing TransformUs    6. Feel confident that the school can support teachers as they adjust to implementing TransformUs 4. Organisational climate: How much do you agree or disagree with the following statements^(c)^:    1. Teachers will be/are expected to use TransformUs strategies    2. Teachers will be/are expected to help the school meet its goals relating to TransformUs    3. Teachers will be/are supported to integrate TransformUs into existing teaching and/or school practices and policies    4. Teachers will be/are supported to deliver TransformUs strategies to students within the school    5. Teachers (will) receive recognition for implementing TransformUs    6. Teachers (will) receive appreciation for implementing TransformUs 5. Decision to register: What made you decide to register for TransformUs? Please select all that apply^(d)^. 6. Encouragement/recommendation from other staff in your school; 7. Awareness/recommendation from another school 8. School board/council and/or parent encouragement 9. To meet physical activity related State/Territory targets 10. Consistent with school policies/school culture 11. To increase school recognition/profile 12. To improve the health and wellbeing of students 13. To improve the mental health of students 14. To improve the educational outcomes of students 15. To help manage the classroom behaviour of students 16. To increase students’ concentration and focus in class 17. To increase the activity levels of students 18. Contribution towards enhancing professional knowledge and/or teaching practice 19. To access teaching and learning resources 20. Other (please specify) 21. Champion: Our school has staff who coordinate the implementation of TransformUs. This means someone who actively and enthusiastically advocates for the use of TransformUs such as a Champion^(e)^ (Yes – it was me, Yes - another teacher, Yes - a team of staff, Other: Please specify, No, Don’t know) 22. External providers and funding: In the past 12 months, has your school received any funding for physical activity related initiatives from any organisations? (yes, no, don’t know)^a^; please give details (please specify) 23. Current practice, school environment. Please indicate (Select strongly disagree, disagree, Neither agree nor disagree, Agree, Strongly agree) how much you agree or disagree with the following statements:     1. Physical activity is a top priority at our school     2. Our school has policies that promote physical activity (e.g., sports uniform, active transport, compulsory sport to year 12)     3. Our school has policies that restrict physical activity (e.g., no eating on the oval, ball free zones)     4. Physical activity often takes a backseat to academic performance     5. Currently my school has…        1. Organised physical activities at lunch that students are interested in        2. Organised physical activities at recess that students are interested in        3. An outdoor environment that promotes movement (e.g., equipment, facilities or line markings such as four square)        4. A classroom environment that promotes movement (e.g., standing desks)        5. Teachers who deliver active lessons (e.g., moving to learn in maths) - not including PE        6. Teachers who deliver active breaks (e.g., breaks from prolonged sitting) - not including PE        7. Teachers who give active homework -not including PE 24. Active lessons. Active lessons are your normal planned lessons, where the delivery method rather than the content is changed. Instead of sitting, to learn, students’ bodies or movement become the vehicle for learning.     1. How confident are you that you could deliver class lessons (excluding PE/sport) that require students to stand up and move around instead of sitting (e.g., active maths lesson)(Please select: Not at all confident, Slightly confident, Moderately confident, Very confident, Extremely confident)     2. Do you deliver class lessons (excluding PE/sport) that require students to stand up and move around instead of sitting (e.g., active maths lesson)? (yes – no)     3. How often? (Please select: Less than once/ month, Once/Month, Once/Fortnight, 1-2 days/week, 3-4 days/week, Once/day, 2 times/day, 3 times/day, 3+ times/day)     4. On average how long per lesson? (Please select: Less than 10mins, 10-20mins, Greater than 20mins)     5. Do you intend to continue to deliver active lessons in the future (yes- no)^(b)^     6. Are you willing to deliver active lessons? (yes – no) 25. Active breaks. Active breaks break up prolonged periods of sitting. Active breaks are short in duration and can be incorporated into any lesson.     1. How confident are you that you could break up prolonged periods of sitting during class time (excluding PE/sport) (Please select: Not at all confident, Slightly confident, Moderately confident, Very confident, Extremely confident)     2. Do you currently break up extended teaching blocks with active breaks (excluding PE/sport)? (yes – no)     3. Do you currently break up extended teaching blocks with active breaks (excluding PE/sport)?     4. Why do you use Active breaks? Please select all that apply.        1. Energise: As an 'Energiser' to break prolonged periods of sitting.        2. Transition: To allow intentional, structured and task orientated movement as students transition between one learning task or phase of the lesson, to the next.        3. Manage: To manage the classroom proactively and positively.        4. Structure: To actively structure the lesson by replacing a traditionally sedentary instructional task with an active version of the same instructional practice.        5. Learn: To introduce, reinforce, consolidate, or demonstrate learning in a visual and physical active way.     5. How often per lesson? (please select: Once Twice, Three times, Four times, Five times, Six times +)     6. Do you intend to continue to deliver active breaks in the future (yes- no)^(b)^     7. Are you willing to deliver active breaks? (yes – no) 26. Active homework. Active homework incorporates a standing and/or moving component within the current homework you set for your students     1. How confident are you that you could provide a standing and/or moving component within the current homework you set for your students? (Please select: Not at all confident, Slightly confident, Moderately confident, Very confident, Extremely confident)     2. Do you currently incorporate a standing and/or moving component within the current homework you set for your students? (yes – no)     3. If yes, what percentage of homework tasks incorporate standing or moving (Please specify)     4. Do you intend to continue to deliver active homework in the future? (yes – no)^(b)^     5. Do you intend to set active homework? (yes – no) 27. Perceptions about active classrooms     1. For each aspect of active teaching and movement integration listed in the table below, indicate how YOU perceive your level of competence (Please select: 1. Very incompetent, 2. Incompetent, 3. Neither, 4. Competent, 5. Very competent)        1. Lesson planning        2. Implementing teaching and learning strategies        3. Managing the class        4. Achieving desired curriculum outcomes        5. Managing curriculum demands     2. Please indicate the degree to which the following factors may act as barriers or inhibit the implementation of active breaks and lessons in your class (Select appropriate category: 1 = No barrier or does not inhibit, 3= moderate barrier, 5 = A major barrier or strongly inhibits)        1. Insufficient training        2. Class size too big        3. Lack of confidence        4. Lack of time        5. Lack of school support        6. Inadequate facilities and equipment        7. Low level of personal interest and enthusiasm        8. Classroom management        9. Litigation concerns        10. Negative student attitude towards active breaks and lessons        11. Demands to teach other curriculum areas        12. Teaching students with additional needs        13. Teaching students with behavioural challenges        14. Other: (please list)     3. Which of the following helped you to implement TransformUs Secondary in your school?^(e)^ Please select all that apply.        1. Participating in a TransformUs Secondary Professional Learning session (online or face-to-face).        2. The ability to adapt the program to my students’ needs (e.g., it was easy for me to modify an Active Break designed for Maths to be suitable for English instead).        3. Being recognised and rewarded for participating in the program (e.g., time taken to undertake TransformUs Professional Learning counted towards yearly requirements).        4. Having a TransformUs champion in my school who promoted and encouraged participation.        5. The resources and lesson plan ideas (e.g., Active Breaks, Active Lesson tasks) and suggested changes to the school environment provided on the website.        6. Utilising a regular teaching team meeting to discuss barriers to implementation and problem-solve together.        7. Utilising a regular teaching team meeting to share and reflect on positive experiences of implementing TransformUs Secondary.        8. Actively involving parents in the program (e.g., encouraging parents to model active learning with their children).        9. Actively involving students in the program (e.g., encouraging students to engage with the TransformUs Secondary peer support resources).        10. Using existing and/or new sources of funding that are available to my school (e.g., to purchase standing desks).        11. Workload was adjusted to allow me to adequately prepare to implement the program.        12. Formal school policies were established in relation to TransformUs Secondary (e.g., daily Active Breaks must be used in each lesson).        13. Others, please mention |
| 1. **Online survey (via Redcap)**   Trial:  ET  Participants:  Consented students  Timepoint:  Baseline  Follow up | 1. Self-reported sociodemographic data: 2. What is your gender? (Female, Male, other, prefer not to say) 3. What is your date of birth? 4. What is your year level? (Year 7,8, 9 and 10) 5. Where were you born? (Australia, England, India, China, New Zealand, Philippines, Vietnam, South Africa, Malaysia, Italy, Other (please specify): 6. How long have you lived in Australia? (please specify: Years, months) 7. What is the main language spoken at home? English, Mandarin, Arabic, Vietnamese, Cantonese, Punjabi, Greek, Italian, Filipino/Tagalog, Hindi, Other (please specify): 8. Youth Activity Profile (YAP)^48^ 9. Class and school engagement (Please select: Not at all true, Not very true, Sort of true, Very true) 10. I try hard to do well in school. 11. I enjoy learning new things in class. 12. When we work on something in class, I feel discouraged. 13. In class, I do just enough to get by. 14. Class is fun. 15. In class, I work as hard as I can. 16. When I’m in class, I feel bad. 17. When I’m in class, I listen very carefully. 18. When I’m in class, I feel worried. 19. When we work on something in class, I get involved. 20. When I’m in class, I think about other things. 21. When we work on something in class, I feel interested. 22. Class is not all that fun for me. 23. When I’m in class, I just act like I’m working. 24. When I’m in class, I feel good. 25. When I’m in class, my mind wanders. 26. When I’m in class, I participate in class discussions. 27. When we work on something in class, I feel bored. 28. I don’t try very hard at school. 29. I pay attention in class. 30. Measure of Adolescent Well-being: EPOCH (Engagement, Perseverance, Optimism, Connectedness, and Happiness) questionnaire.   (Please select: Almost Never, Sometimes, Often, Very Often, Almost Always)   1. When something good happens to me, I have people who I like to share the good news with. 2. I finish whatever I begin. 3. I am optimistic about my future. 4. I feel happy. 5. When I do an activity, I enjoy it so much that I lose track of time. 6. I have a lot of fun. 7. I get completely absorbed in what I am doing. 8. I love life. 9. I keep at my schoolwork until I am done with it. 10. When I have a problem, I have someone who will be there for me. 11. I get so involved in activities that I forget about everything else.   (Please select: Not at all like me, A little like me, Somewhat like me, Mostly like me, Very much like me)   1. When I am learning something new, I lose track of how much time has passed. 2. In uncertain times, I expect the best. 3. There are people in my life who really care about me. 4. I think good things are going to happen to me. 5. I have friends that I really care about. 6. Once I make a plan to get something done, I stick to it. 7. I believe that things will work out, no matter how difficult they seem. 8. I am a hard worker. 9. I am a cheerful person.   5. Sleep (Please indicate: HH:MM): ____ : ____)   1. What time do you go to bed on school nights? 2. What time do you go to bed on weekends? 3. What time do you get out of bed on school days (weekdays)? 4. What time do you get out of bed on weekends? 5. How long do you usually sleep at night on school nights (weeknights)? 6. How long do you usually sleep at night on weekends? 7. Adolescent Sleep Wake Scale (please select: Never, Once in a while, Sometimes, Quite often, Frequently, if not always, Always) 8. When it’s time to go to bed, I want to stay up and do other things 9. In general, I am ready for bed at bedtime 10. In general, I try to “put off” or delay going to bed 11. When it’s time to go to sleep, I have trouble settling down 12. In general, I need help getting to sleep (for example, I need to listen to music, watch TV, take medication, or have someone else in the bed with me) 13. After waking up during the night, I have trouble going back to sleep 14. After waking up during the night, I have trouble getting comfortable 15. After waking up during the night, I need help to go back to sleep (for example: I need to watch TV, read, or sleep with another person) 16. In the morning, I wake up and feel ready to get up for the day 17. In the morning, I wake up feeling rested and alert 18. When it’s time to go to bed, I want to stay up and do other things 19. In general, I am ready for bed at bedtime 20. In general, I try to “put off” or delay going to bed 21. When it’s time to go to sleep, I have trouble settling down 22. In general, I need help getting to sleep (for example, I need to listen to music, watch TV, take medication, or have someone else in the bed with me) 23. After waking up during the night, I have trouble going back to sleep 24. After waking up during the night, I have trouble getting comfortable 25. After waking up during the night, I need help to go back to sleep (for example: I need to watch TV, read, or sleep with another person) 26. In the morning, I wake up and feel ready to get up for the day 27. In the morning, I wake up feeling rested and alert 28. Current practice: Currently, my school has…(Please select: Doesn’t have, Strongly disagree, Disagree, Neither agree or disagree, Agree, Strongly agree) 29. Organised physical activities at lunch that I am interested in 30. Organised physical activities at recess that I am interested in 31. An outdoor environment that promotes movement (e.g., equipment or facilities) 32. A classroom environment that promotes movement (e.g., standing desks) 33. Teachers who deliver active lessons (e.g., moving to learn in maths) -not including PE 34. Teachers who deliver active breaks (e.g., breaks from prolonged sitting) -not including PE 35. Teachers who give active homework -not including PE |
| 1. **Classroom observation**   Trial:  ET  Participants:  Students attending to classes of teachers who attended professional learning  Timepoint:  Baseline  Follow up | The observation will assess students:   1. Behaviours (Focused on-task, Passive off-task, Off-task and Absent) 2. Movement behaviour (Sitting, Standing, Walking slow, Walking fast, Moving fast [i.e., jumping]) 3. Setting (Independent, Paired, Group, Teacher-led, Test, Other) |
| 1. **Observed physical activity during recess and lunch**   Trial:  ET  Participants:  Consented students  Timepoint:  Baseline  Follow up | SOPLAY codes and recording^47^   1. Reliability: Circle 'NO" unless you are the second observer and your data will serve as a reliability measure. 2. Temperature: Enter Fahrenheit temperature at the start of the observation period.   Period: Circle a number to designate whether observations were made before school (BS), at lunch time (L), or after school (AS).   1. Start time: Enter the start time (2400 hours) of the sweep for that designated area. 2. Area: Refers to the number of a previously designated School Target Area (see school map). If necessary, add an additional area, describe it, and give it a new number. 3. Condition: Circle No or Yes to describe specific conditions for each designated observation area. If a Target Area is inaccessible (A=N), do not code the other four conditions. A = Area is accessible (e.g., not locked or rented to others)   U = Area is usable for physical activity (e.g., is not excessively wet or windy); S = Area is supervised by designated school or adjunct (e.g., YMCA) personnel (e.g., teachers, playground supervisors, volunteers). The supervisor must be in or adjacent to that specific area (i.e., available to direct students and respond to emergencies), but does not have to be instructing, officiating, or organizing activities; O = Organized physical activity (i.e., scheduled, with leadership by school or agency personnel apparent) is occurring in the area (e.g., intramurals, interscholastic practices, fitness stations); E = Equipment provided by the school or other agency is present (e.g., balls, jump, ropes). Do not code 'YES' if the only equipment is permanent (e.g., basketball hoops) or is owned by students themselves.   1. S = Sedentary; W = Walking; V = Very Active 2. Activity: Enter the activity code (or name) for the most prominent physical activity that girls and boys are participating in within designated area. |
| 1. **Text messages**   Trial:  IT- ET  Participants:  Participants who agree to take part in the research component  Timepoint:  During the Evaluation period (up to three times per term) | Which of the following TransformUs strategies have you implemented in the last week?   1. Active Lessons 2. Active breaks 3. Organised activities at recess and lunch 4. Making changes to the school environment (indoor or outdoor) 5. Active homework 6. Peer-support 7. Health lessons 8. None of the above   Respond with relevant numbers, e.g., if you have use Active Lessons and Active Breaks, respond with "1, 2". |
| 1. **Qualitative assessment**   Trial:  IT (Focus group)  ET  (interview)  Participants:  Teachers and school staff who agree to take part in the research component (sub-sample)  Timepoint:  Follow up | Interview/focus group schedule  You have been invited to participate in this interview/focus group as you work at one of the schools currently implementing TransformUs. This discussion should last no more than 30 minutes (interviews) and ~2 hours (focus group). Questions will relate to your experiences of delivering the program, including any barriers or enablers you might have faced. There are no right or wrong answers, so please speak freely and honestly about your experience to date. This interview/focus group will be audio-recorded, transcribed and coded anonymously for analysis. Your name will not be used in any of the published material and your responses will remain unidentifiable. Note: questions 1 to 23 and 25 to 28 will be adapted or deleted for the focus group context.  Researcher: I want to start by asking you a few basic questions about yourself.  Descriptive   1. Which school do you currently work at? (Ask if it has a campus) 2. What is your current job role (if in the class what subjects do you currently teach?) 3. How long have you held this position?   TransformUs involves strategies in the classroom (such as standing lessons and active breaks), changes to the classroom or broader school environment and activities at recess and lunch.  Reach  4. How did you hear about TransformUs?  5. What do you think is the most effective way for a program such as TransformUs to be promoted within your school? (prompt, to maximise the likelihood of uptake)  6. Have you accessed any of the TransformUs online PD in the members area of the website (e.g., help guides on the different strategies)?  7. Have you looked at any of the online resources in the members area?If yes, what aspects did you access? How did you find them?  8. Are you delivering TransformUs?  9. What has the uptake of the program been like across your school? Prompt if needed (Please elaborate)  10. What characteristics do you believe are associated with implementing or not implementing the program? (prompt, in terms of teaching style, subject etc.)  Effectiveness  11. Overall, would you say that the program has been successful in your school? (prompt if needed, how do you define success?) If yes, why? If no, why not?  12. Do you think TransformUs has had any positive or negative impact on your school or the students? (If yes, what impact? If no, why not?)  13. Do you think TransformUs has had any impact, positive or negative, on your teaching practices? ( If yes, what impact? If no, why not?)  14. What are the strengths and challenges of the program?  15. Are you aware of any changes within the school, positive or negative, regarding physical activity and sedentary behaviour practices and/or policies? (prompt if needed, changes to culture or norms re PA and/or SB) (If yes, please describe?, If no, what would facilitate positive changes?)  Adoption  16. What influenced your decision to register/adopt the program? (positive and negative)(Prompt if needed: Teacher recommendation, Impact of DET endorsement, Points for CPD, Perceived importance of increasing PA and reducing SB, Perceived outcomes for the school/teachers/students, Perceived need for TransformUs, Perceived benefits of TransformUs  17. What are the major barriers to adoption of programs in schools in general? (prompt if needed, what would overcome those barriers?)  18. Do you think there are any similarities or differences between schools that would choose or choose not to implement it? (prompt if needed, please explain)  Implementation  Dose/Fidelity  19. Which of the TransformUs strategies have you been using over the past 12 months? (Why did you chose those strategies? Did you implement them “successfully”? (prompt if needed, how define success), Why did you not implement some strategies?  20. Were there any barriers or facilitators to your delivery of the program? (Ask this for each of the strategies they have been using <answered in question 1>) prompt if needed, did they change over time?)  21. Have you undertaken any strategies to overcome challenges to delivery? (If yes, describe?, If no, why not OR what could have assisted you in overcoming them?)  22. Have you shared, or have others shared, techniques for implementing the program? (If so, can you describe these?)  Adaptation  23. Did you make any adaptations to the program in order to implement it? (prompt if needed, to suit teaching practice, planned lessons, student’s learning styles)If yes, can you describe them? )  Implementation strategies (focus group only)  24. Please mention strategies that support you in implementing and maintaining the program in your school.  a. Who implement/provide it?  b. How is it implemented/provided (action)?  c. What is this purpose (action target)? (i.e., to support implementation or maintenance or both?)  d. When is it implemented/provided (temporality)?  e. How frequent and how long is it implemented/provided (temporality)?  f. What does it impact (outcome affected)?  g. Why do you implement/provide/need it? What barriers to address (justification)?  Maintenance  25. To what extent do you think the program is embedded as part of your school’s routine teaching practices and/or school policies? (It is embedded, what facilitated this?, It isn’t embedded, what were the barriers?)  26. Are you planning to continue delivering the program after this interview? (If yes, what will enable you to do this?, If no, why not?)  Recommendations  27. Based on your experiences over the last year, can you recommend ways to:  (select as appropriate based on previous answers: Increase uptake of the program among teachers and/or schools; Make the program more likely to have positive outcomes  (prompt, on children/teachers/school); Improve implementation of the program in practice; Increase the likelihood it becomes embedded as part of the schools' routine practice.  28. Do you have any further comments you would like to make that we haven’t covered? |

(*) The TransformUs website includes, in addition to TransformUs Secondary, Primary, and All abilities programs; only relevant information regarding to TransformUs Secondary will be used for the purpose of this study). Legend: IT, implementation trial; ET, effectiveness trial.

a Baseline only; b Follow up only; c Effectiveness Trial ‘waitlist control’ participants won’t be asked; d Baseline only; effectiveness Trial ‘waitlist control’ participants won’t be asked; e Follow up only; effectiveness Trial ‘waitlist control’ participants won’t be asked.

**Online Supplementary File 5: Students and teacher consent forms.**

1. ***Effectiveness trial: staff consent form***

**School Staff Consent Form**

1. I have read and I understand the attached Plain Language Statement*.*
2. I have been given a copy of the Plain Language Statement and Consent Form to keep.
3. I freely agree to participate in this project and the evaluation as described in the Plain Language Statement.
4. I understand that I may not be invited to complete an interview.
5. I understand that the interviews with the researchers will be audio recorded.
6. Aggregated results will be used for research purposes and may be reported in scientific and academic journals and conference meetings.
7. The researcher has agreed not to reveal my identity and personal details if information about this project is published or presented in any public form.

My name ………………………………………………………

My role ………………………………………………………

My mobile number: …………………………………………………….

My email address: …………………………………………………….

**By checking this box, I indicate that I have read the Plain Language Statement and consent to take part in this project.**

1. ***Effectiveness trial: student/parent consent form***

**Student/Parent Consent Form**

1. I have read and I understand the attached Plain Language Statement*.*
2. I have been given a copy of the Plain Language Statement and Consent Form to keep.
3. I freely agree to participate in this project and the evaluation as described in the Plain Language Statement.
4. Aggregated results will be used for research purposes and may be reported in scientific and academic journals and conference meetings.
5. The researcher has agreed not to reveal the participants’ identities and personal details if information about this project is published or presented in any public form.

Student consent

**I, the student, indicate that I have read the Plain Language Statement and consent to the three main assessments described above (accelerometer, survey, and classroom observation).**

**Yes**

**No**

**I, the student, indicate that I have read the Plain Language Statement and consent to the two optional assessment described above (inclinometer and focus).**

**Yes**

**No**

**Parent/guardian consent**

**I, the parent/guardian, indicate that I have read the Plain Language Statement and consent for my child to have the three main assessments described above (accelerometer, survey, and classroom observation).**

**Yes**

**No**

**I, the parent/guardian, indicate that I have read the Plain Language Statement and consent for my child to have the two optional assessment described above (inclinometer and focus group).**

**Yes**

**No**

1. **Implementation trial: staff consent form**

**Implementation Trial Consent Form**

1. I have read and I understand the attached Plain Language Statement*.*
2. I have been given a copy of the Plain Language Statement and Consent Form to keep.
3. I freely agree to participate in this project and the evaluation as described in the Plain Language Statement.
4. I understand that I may not be invited to complete an interview or focus group.
5. I understand that the interviews/focus groups with the researchers will be audio recorded.
6. Aggregated results will be used for research purposes and may be reported in scientific and academic journals and conference meetings.
7. The researcher has agreed not to reveal my identity and personal details if information about this project is published or presented in any public form.

My name ………………………………………………………

My role ………………………………………………………

My mobile number: …………………………………………………….

My email address: …………………………………………………….

**By checking this box, I indicate that I have read the Plain Language Statement and consent to take part in this project.**
