# Supplementary material for: Protocol of the TransformUs Secondary schools program: a type II hybrid implementation-effectiveness trial to increase adolescents’ physical activity and reduce sedentary time in secondary schools
Source: BMJ Open. 2025 Feb 10;15(2):e090468. doi: 10.1136/bmjopen-2024-090468 (PMC11815412; doi:10.1136/bmjopen-2024-090468)
Supplement: Supplementary file 2 [file bmjopen-2024-090468.reviewer_comments.pdf]

PEER REVIEW HISTORY

BMJ Open publishes all reviews undertaken for accepted manuscripts. Reviewers are asked to complete a checklist review form and are provided with free text boxes to elaborate on their assessment. These free text comments are reproduced below.

ARTICLE DETAILS

Title (Provisional)

Protocol of the TransformUs Secondary Schools program: A Type II hybrid implementation-effectiveness trial to increase adolescents' physical activity and reduce sedentary time in secondary schools

Authors

Contardo Ayala, , Ana María; Lander, Natalie; Mazzoli, Emiliano; Timperio, Anna; Koorts, Harriet; Ridgers, Nicola D.; Abbott, Gavin; Lubans, David Revalds; Salmon, Jo

---

VERSION 1 - REVIEW

**Reviewer**	**1**
**Name**	**Muller, Andre**
**Affiliation**	**National University of Singapore, Saw Swee Hock School of Public Health**
**Date**	**12-Sep-2024**
**COI**	**None**

Dear authors,

Thank you for this excellent piece. I am very happy to see this great nationwide implementation effort. This study will hopefully provide guidance on translating small-scale trials to large-scale rollouts. It is great to see how you implement strategies that may truly tackle key ecological factors to generate an activity-supporting ecosystem.

As I teach university students, I incorporate movement breaks regularly; your idea to even incorporate movement prompts into assignments and other asynchronous learning tasks is inspiring. I also appreciate that context-related customizations were incorporated; it's not a one-size-fits-all approach.

The effectiveness trial has been designed well. I appreciate the different strata to account for various schools and contexts. I imagine that recruitment and follow-up are critical elements that can make or break the study. I hope it all goes well and that you can achieve the required numbers.

Wishing you all the best for the implementation. I hope this work will bring positive changes to the population in both the short and long term.

I have no major suggestions but wonder whether the following could be reflected upon: Why is activity so low and sedentary behavior so high among secondary school kids in Australia? Having visited the country this year, I had the impression that kids are often out and about. After all, Australia is known for its outdoor lifestyle. From my interactions with locals, I also learned that academic pressure is not as high as in many parts of Asia (where I am based). Perhaps a short explanation would be helpful.

Reviewer **2**

Name **Machado-Rodrigues, Aristides M.**

Affiliation **Universidade de Coimbra, Faculty of Sport Sciences and Physical Education**

Date **12-Sep-2024**

COI **None**

Comments to the authors:

Comment 1. The purpose of the present article was to describe the protocol for TransformUs Secondary in relation to implementation and scale-up across Australia, and real-world effectiveness of the intervention on adolescents' physical activity and sedentary time, as well as sitting time, sleep, wellbeing, and class and school engagement. The role of lifestyle among youth is certainly a topic of great importance as we move toward a better understanding of the role that behavioural and socio-cultural factors have on health of our paediatric population.

Comment 2. In general, the abstract summarizes the paper properly; however, there are some specific details that might be help the reader to better understand the analytical approach.

Comment 3. In the introduction section, the purpose of the study could be stated clearer. Furthermore, the reason for this focus, despite of acceptable, could also include other important arguments to emphasise the need of further studies in that specific topic. Furthermore, it would be important to clearly delimitate conceptual and instrumental issues on that preliminary section of the manuscript.

Comment 4. The methods section is somewhat unbalanced in terms of its content when viewed in a holistic perspective of that manuscript. For example, the sub-heading Student level outcomes could be better structured.

Comment 5: Furthermore, there are some methodological decisions that must be clarified (for example, among others, how data may be generalized??).

Comment 6: According to the specific framework of the present study, the manuscript would benefit by including some potential results based on the decisions and the context where data will be collected.

Comment 7: The data analyses sub-section is stated pragmatically.

Comment 8: The references are, in general, appropriate. However, they could be, in some issues , updated.

VERSION 1 - AUTHOR RESPONSE

	REVIEW COMMENT	RESPONSE TO COMMENT	CHANGE MADE TO MANUSCRIPT (new or updated section highlighted in yellow)
**Reviewer#1 Dr. Andre Muller, National University of Singapore**			
**4**	Thank you for this excellent piece. I am very happy to see this great nationwide implementation effort. This study will hopefully provide guidance on translating small-scale trials to large-scale rollouts. It is great to see how you implement strategies that may truly tackle key ecological factors to generate an activity-supporting ecosystem.<br>As I teach university students, I incorporate movement breaks regularly; your idea to even incorporate movement prompts into assignments	Thank you very much for your kind and supportive feedback. We are thrilled that you see the value in this nationwide implementation and its potential to help bring smaller trials to a larger scale.<br>We agree that recruitment and follow-up will be crucial for the study's success, and we have put several measures in place to help with both.	N/A

	and other asynchronous learning tasks is inspiring. I also appreciate that context-related customizations were incorporated; it's not a one-size-fits-all approach. The effectiveness trial has been designed well. I appreciate the different strata to account for various schools and contexts. I imagine that recruitment and follow-up are critical elements that can make or break the study. I hope it all goes well and that you can achieve the required numbers. Wishing you all the best for the implementation. I hope this work will bring positive changes to the population in both the short and long term.		
**5**	I have no major suggestions but wonder whether the following could be reflected upon: Why is activity so low and sedentary behavior so high among secondary school kids in Australia? Having visited the country this year, I had the impression that kids are often out and about. After all, Australia is known for its outdoor lifestyle. From my interactions with locals, I also learned that academic pressure is not as high as in many parts of Asia (where I am based). Perhaps a short explanation would be helpful.	While Australia has a strong outdoor activity culture, research consistently shows that physical activity levels among secondary school students remain low, with high rates of sedentary behaviour. Possible contributing factors include the increase in screen-based activities (such as social media and gaming) (Thomas et al., 2020), the structured nature of school schedules that limit movement during school hours (Salmon et al. l, 2020), and, in some areas, limited access to safe outdoor spaces after school.<br>We agree that a brief explanation of these factors could help contextualise the study and have included a short reflection	See introduction section:<br><br>*While Australia offers some opportunities for sport and physical activity participation,*[7] research consistently shows that physical activity levels among secondary school students remain low, with only 8% of secondary school students meeting physical activity guidelines and more than two-thirds of their waking hours spent in sedentary behaviour8.

		in the introduction to address this.	
**Reviewer #2 Dr. Aristides M. Machado-Rodrigues, Universidade de Coimbra**			
**6**	The purpose of the present article was to describe the protocol for TransformUs Secondary in relation to implementation and scale-up across Australia, and real-world effectiveness of the intervention on adolescents' physical activity and sedentary time, as well as sitting time, sleep, wellbeing, and class and school engagement. The role of lifestyle among youth is certainly a topic of great importance as we move toward a better understanding of the role that behavioural and socio-cultural factors have on health of our paediatric population.	Thank you for reviewing our manuscript.	N/A
**7**	In general, the abstract summarizes the paper properly; however, there are some specific details that might be help the reader to better understand the analytical approach.	We revised the abstract to provide a clearer explanation of our analytical methods to enhance understanding.	Abstract:<br><br>Implementation outcomes will be evaluated using the RE-AIM framework (reach, adoption, implementation, and maintenance), with data collected at school and teacher levels through the TransformUs website (website analytics), surveys, text messages, and interviews with teachers and key organisation representatives. *Descriptive statistics will summarise quantitative data, with regression analyses examining associations between implementation strategies and outcomes. Implementation levels will be classified as low, moderate, or high based on the extent of intervention delivery. Qualitative data will be thematically analysed.*

			Effectiveness outcomes will be assessed in a pragmatic 1:1 waitlist control design across 10 Victorian secondary schools, targeting 600 Year 7-10 students (ages 12-16). Primary outcomes are physical activity and sedentary time, measured via accelerometry, with secondary outcomes including sleep, wellbeing, class and school engagement (on-task behaviour, school attendance), and sitting time (assessed with posture monitors). *Descriptive analyses will summarise students' demographics, physical activity, sedentary behaviour, and engagement, while mixed models will evaluate intervention effects on these outcomes, adjusted for confounders. Additionally, qualitative data will be thematically analysed using deductive and inductive coding in NVivo.*
**8**	In the introduction section, the purpose of the study could be stated clearer. Furthermore, the reason for this focus, despite of acceptable, could also include other important arguments to emphasise the need of further studies in that specific topic. Furthermore, it would be important to clearly delimitate conceptual and instrumental issues on that preliminary section of the manuscript.	We have clarified the study's purpose in the introduction, providing a more concise statement of our objectives and rationale. We have also expanded on the importance of investigating adolescent physical activity and sedentary behaviour in secondary schools, highlighting gaps in existing research and emphasising the broader implications for adolescent health and educational outcomes.	See the introduction section:<br>Few effective school-based physical activity programs have been implemented at scale in secondary schools[14], and these initiatives primarily concentrate *on enhancing physical education sessions[15-17], and prioritising educational strategies[18] over whole-of-school interventions that incorporate movement throughout the day. This is despite adolescents spending a substantial portion of their school hours in the classroom[19], which remains underutilised in terms of active learning. There is a need to explore the implementation of whole-of-school interventions that can support physical activity not only during physical education but also within core curriculum areas, such as maths, science,*

			and English, and also, there is potential to adapt and test implementation and effectiveness of successfully scaled primary school physical activity programs in secondary schools[20].<br><br>*This paper provides a rationale and description of the TransformUs Secondary. The study will include both an implementation trial, aimed at determining the implementation of TransformUs Secondary offered at scale to all secondary schools in Australia (Implementation trial), and an effectiveness trial to assess the impact of the intervention on movement behaviours, cognitive/academic outcomes, and psychological well-being among adolescents and teachers attending Victorian secondary schools. (Effectiveness Trial).*
**9**	The methods section is somewhat unbalanced in terms of its content when viewed in a holistic perspective of that manuscript. For example, the sub-heading Student level outcomes could be better structured.	We restructured the Student Level Outcomes subsection to enhance clarity and logical flow. We also restructured the teacher and school-level outcomes sections for both trials.	Please see student level outcome section on page 16, teacher-level outcomes on pages #11-12 (Implementation trial) and #16-19 (effectiveness trial), and school-level outcomes on page#20.
**10**	Furthermore, there are some methodological decisions that must be clarified (for example, among others, how data may be generalized??).	In response to your query about the generalizability of the results, we have added clarifications in the Methods section regarding the sample size and power calculation for both the implementation and effectiveness trials.	See Sample size and power calculation (implementation trial) section:<br>*Making the intervention available to all schools enhances the generalisation of results by reaching diverse student populations, ensuring consistent implementation, and testing in varied real-world contexts (across school types and states and territories). This approach increases the sample size, allows for replication of outcomes, and*

Commented [AC1]: Or maybe in the recruitment section?

			provides valuable feedback for continuous improvement.<br><br>See Sample size and power calculation (effectiveness trial) section:<br><br>*Including an effectiveness trial that is accessible to all schools in Victoria enhances the generalizability of the results by incorporating diverse student populations representing various educational contexts, needs, and resources. This broad approach improves the reliability of findings, reflecting how the intervention performs across different demographics and settings, making the results more applicable to other schools in the state. However, using a subsample may limit generalisation as it may not fully represent the student population's diversity. Smaller or more localised samples could be influenced by specific local conditions, reducing the ability to make broader conclusions. While subsamples offer valuable insights, they may lack the comprehensiveness needed for strong generalisations, which we have acknowledged as a limitation.*
**11**	According to the specific framework of the present study, the manuscript would benefit by including some potential results based on the decisions and the context where data will be collected.	Thank you for this suggestion. As this is a protocol manuscript, no results are available since the data collection has not yet commenced. In accordance with BMJ Open guidelines for protocol publications, a discussion or potential results section is not required, as the focus is on detailing the study design and methods to	N/A

		ensure transparency and reproducibility.<br><br>We have, however, described the framework and context of the study, including how the implementation and effectiveness outcomes will be evaluated, to give readers a clear understanding of the anticipated scope and significance of the findings	
**12**	The data analyses sub-section is stated pragmatically.	Thank you for your observation. The data analysis methods were intentionally presented pragmatically to ensure clarity and applicability, reflecting the study's hybrid implementation-effectiveness design. We have detailed the planned descriptive statistics, regression analyses, and mixed models to address the study’s objectives. If further elaboration or theoretical grounding is required, we would happily incorporate this into the manuscript.	N/A
13	The references are, in general, appropriate. However, they could be, in some issues , updated.	We have reviewed and updated the references where more recent or relevant studies are available.<br><br>The updated references are: 15, 23, 39	Please see the reference list.

VERSION 2 - REVIEW

Reviewer **2**

Name **Machado-Rodrigues, Aristides M.**

Affiliation **Universidade de Coimbra, Faculty of Sport Sciences and Physical Education**

Date **20-Dec-2024**

COI

Comments to the authors:

Comment 1. The purpose of the present article was to describe the protocol for TransformUs Secondary in relation to implementation and scale-up across Australia, and real-world effectiveness of the intervention on adolescents' physical activity and sedentary time, as well as sitting time, sleep, wellbeing, and class and school engagement. The authors did an important effort to improve the manuscript, and therefore, it should be clearly emphasized. Very well done!

General Comment 2. In general, the abstract summarizes the paper properly.

General Comment 3. At the introduction section, the purpose of the study was stated clearly, as well as the reason for this focus.

Comment 4. The authors did a sounds job of outlining the protocol in the methods section.

General Comment 5: In general, the quality of writing is satisfactory but could be clearly improved.

General Comment 6: The references are ok.
